# Nanoparticle-mediated mRNA delivery for cancer, autoimmunity, and genetic diseases: a rapid review

**DOI:** 10.3389/fddev.2026.1793322

**Published:** 2026-04-01

**Authors:** Okechukwu Paul-Chima Ugwu, Fabian C. Ogenyi, Mariam Basajja, Chinyere N. Ugwu, Mundu M. Mustafa, Michael Ben Okon

**Affiliations:** 1 Department of Publication and Extension, Kampala International University, Kampala, Uganda; 2 Healthcare and Data management, Leiden University, Leiden, Netherlands; 3 Renewable Energy Systems, Kampala International University, Kampala, Uganda

**Keywords:** autoimmunity, cancer immunotherapy, circular RNA, genetic therapy, lipid nanoparticles, messenger RNA, non-viral delivery

## Abstract

**Introduction:**

Messenger RNA (mRNA) therapeutics have advanced from experimental platforms to clinical application, driven largely by the success of lipid nanoparticle (LNP)-based COVID-19 vaccines. Building on this progress, nanoparticle-mediated mRNA delivery is being extended to non-infectious indications, including oncology, autoimmune disorders, and inherited diseases. However, challenges such as extrahepatic targeting, endosomal escape, repeat‐dose immunogenicity, thermostability, and scalable manufacturing remain significant barriers to translation.

**Methods:**

A rapid review of peer-reviewed studies and registered clinical trials published between January 2020 and October 2025 was conducted. Searches were performed in PubMed, Scopus, Web of Science Core Collection, and ClinicalTrials.gov using combined terms related to RNA modality and nanoparticle delivery. Eligible studies focused on non-viral nanoparticle platforms for therapeutic, non‐infectious mRNA delivery, including applications in protein replacement, genome editing, and immune modulation. Screening yielded 15 studies for inclusion.

**Results:**

LNPs remain the most clinically advanced platform for therapeutic mRNA delivery. At the same time, polymeric, peptide‐based, exosome-inspired, and hybrid nanoparticle systems are expanding the delivery landscape. Emerging RNA formats, including self-amplifying RNA and circular RNA, show potential to prolong expression at lower doses. Clinically, individualized mRNA neoantigen therapy (mRNA-4157/V940) combined with pembrolizumab reduced recurrence risk by approximately 49% in high-risk melanoma in the KEYNOTE-942 phase 2b trial, supporting phase 3 development. In cystic fibrosis, inhaled CFTR mRNA (ARCT-032) advanced to phase 2 after early phase 1 data demonstrated safety and tolerability.

**Discussion:**

Evidence for non-viral nanoparticle‐mediated mRNA therapeutics is strong in preclinical research and increasingly promising in clinical applications beyond vaccinology. While LNPs dominate current translation, alternative carriers and improved RNA formats may broaden tissue targeting and therapeutic durability. Advances in biodegradable ionisable lipids, organ-selective LNPs, and lyophilised or solid formulations are being developed to address persistent delivery and manufacturing constraints. As the field matures, regulatory and policy frameworks will need to align with therapeutic endpoints and support long-term safety monitoring.

## Highlights

- Platform: LNPs lead clinical translation; polymeric, peptide, hybrid, and exosome-inspired carriers provide complementary approaches.
- Clinical signal: mRNA-4157/V940 plus pembrolizumab reduced melanoma recurrence or death by approximately 49% in phase 2b; phase-3 trials are underway.
- Autoimmunity: Tolerogenic LNP-mRNA induces antigen-specific tolerance in EAE; splenic targeting is a key design axis.
- Genetic disease: Inhaled CFTR mRNA has advanced to phase 2; efficacy requires confirmation in sustained, appropriately powered studies.
- Evidence gaps: Extrahepatic targeting, endosomal escape, repeat-dose safety, and thermostability remain limiting but addressable engineering challenges.

## 1 Introduction

**FIGURE 1:**
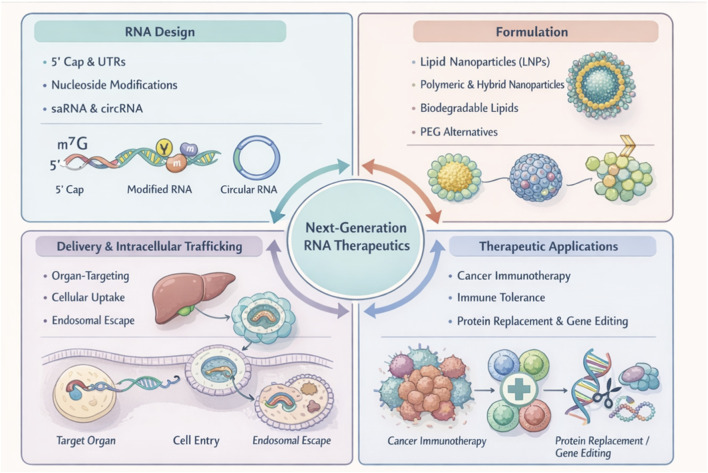
Key design dimensions for next-generation RNA therapeutics. Schematic overview of four interdependent modules that govern RNA therapeutic performance: (i) RNA design, including 5′cap and untranslated regions (UTRs), nucleoside modification, and alternative RNA modalities such as self-amplifying RNA (saRNA) and circular RNA (circRNA); (ii) formulation, encompassing lipid nanoparticles (LNPs), polymeric or hybrid nanoparticles, biodegradable lipid components, and polyethylene glycol (PEG) alternatives to optimise stability and biocompatibility; (iii) delivery and intracellular trafficking, highlighting organ-selective targeting and cellular entry/processing steps; and (iv) therapeutic modules, illustrating representative applications including cancer immunotherapy, antigen-specific immune tolerance, and protein replacement or genome/protein editing. Abbreviations: UTR, untranslated region; saRNA, self-amplifying RNA; circRNA, circular RNA; LNP, lipid nanoparticle; PEG, polyethylene glycol.

Messenger RNA (mRNA) therapeutics have evolved from a conceptual tool for studying gene expression into a clinically validated modality, as illustrated in Figure 1 (Fei et al., 2024; Simonsen, 2024). The rapid development and high effectiveness of LNP-formulated mRNA vaccines during the COVID-19 pandemic demonstrated the feasibility of scalable nucleic-acid medicine manufacture and clarified key regulatory considerations (Zhang et al., 2024; Chatterjee et al., 2024). These advances established mRNA as a broadly applicable therapeutic approach characterised by rapid design, versatile payload encoding, and transient expression without genomic integration (Wang X. et al., 2025).

The field is now expanding beyond prophylaxis to therapeutics, including tumour-specific mRNA vaccines encoding personalised neoantigens in combination with immune checkpoint inhibitors; tolerogenic mRNA programmes that reprogramme immune responses in autoimmune disease; and transient mRNA delivery for protein replacement or genome editing in inherited disorders (Casmil et al., 2025; Vallet and Vignuzzi, 2025). This shift reframes mRNA delivery from pathogen-directed immune stimulation towards disease-context-specific immune modulation and restoration of physiological function (Wang Y. et al., 2025).

Delivery remains the principal determinant of therapeutic performance. Naked mRNA is intrinsically unstable, susceptible to enzymatic degradation, and inefficiently internalised by cells (Shen et al., 2023). Non-viral nanoparticles particularly LNPs have therefore become the leading clinical delivery strategy because they encapsulate and protect mRNA and support cytosolic delivery with negligible risk of insertional mutagenesis (Gabelmann et al., 2025). Their modularity enables optimisation of lipid composition, surface chemistry, charge, and size to tune biodistribution, cellular uptake, endosomal escape, and immunostimulatory profile.

Despite these advances, major obstacles remain to extending mRNA nanotherapeutics beyond hepatotropic delivery and vaccine use (Yu et al., 2025). Most conventional LNPs accumulate in the liver, in part due to adsorption of apolipoprotein E and subsequent uptake pathways, which limits access to extrahepatic targets. Addressing this constraint requires rational lipid design, control of biomolecular corona formation, ligand conjugation, and route optimisation to enhance delivery to tissues such as lung, spleen, and the tumour microenvironment (Pastre et al., 2025). Endosomal escape is an additional bottleneck: only a small proportion of internalised mRNA typically reaches the cytosol, limiting efficiency and therapeutic index. Emerging approaches including self-amplifying RNA (saRNA), circular RNA (circRNA), and biodegradable or organ-selective ionisable lipids aim to improve durability, safety, and biodistribution (Paroor et al., 2025). Collectively, these developments support mRNA as a programmable platform for oncology, autoimmunity, and genetic medicine (Wu et al., 2024).

### 1.1 AIM/objective

This rapid review synthesises preclinical and clinical evidence (2020–2025) on nanoparticle-mediated mRNA delivery beyond infectious-disease prophylactic vaccines, focusing on (i) delivery platform advances and mechanistic enablers (biodistribution, cellular uptake, endosomal escape, and stability), (ii) therapeutic applications in oncology, autoimmunity, and genetic disease, and (iii) translational barriers relevant to clinical development, manufacturing, and regulation.

## 2 Methods

### 2.1 Search strategy and databases

A rapid, scoping-style review was conducted in line with PRISMA 2020 and PRISMA-ScR guidance (Tricco et al., 2018), adapted for expedited synthesis. PubMed, Scopus, Web of Science Core Collection, and ClinicalTrials.gov were searched for records published or registered between 1 January 2020 and 30 October 2025.

Search strings combined controlled vocabulary and free-text terms using Boolean operators: (“messenger RNA” OR mRNA OR “self-amplifying RNA” OR saRNA OR “circular RNA” OR circRNA) AND (“nanoparticle” OR “lipid nanoparticle” OR LNP OR “non-viral vector”) AND (“cancer” OR “autoimmunity” OR “genetic disease” OR “protein replacement” OR “gene editing”).

Searches were restricted to English-language peer-reviewed articles and completed or active clinical trials. Reference lists of eligible papers were screened to identify additional studies.

## 3 Inclusion and exclusion criteria

### 3.1 Inclusion criteria

1. Experimental or clinical studies describing nanoparticle-mediated mRNA delivery for therapeutic (non-infectious) indications.
2. Reports addressing mechanistic, formulation, or translational aspects (e.g., endosomal escape, tissue targeting, stability).
3. Preclinical animal studies, *in vitro* mechanistic studies, and early-phase clinical trials (phases 1–3).
4. Systematic reviews/meta-analyses (2020–2025) providing aggregated data relevant to therapeutics.

### 3.2 Exclusion criteria

1. Vaccine studies limited to infectious-disease prophylaxis without a therapeutic intent.
2. Non-mRNA nucleic acids (e.g., siRNA, DNA, ASOs) unless used as comparators.
3. Conference abstracts, editorials, and commentaries lacking primary data.
4. Non-English literature or inaccessible full texts.

### 3.3 Study selection and screening flow

**FIGURE 2:**
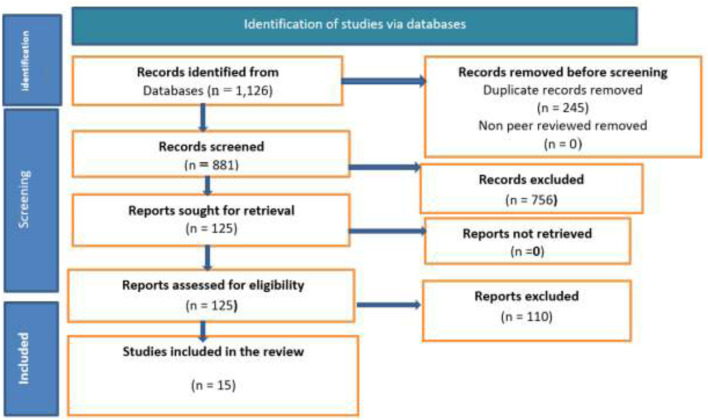
PRISMA flow diagram of study selection. Records were identified through database searching (n = 1,126). After removal of duplicates (n = 245) and non–peer-reviewed records (n = 0), 881 records were screened, of which 756 were excluded. Full-text reports were sought for retrieval (n = 125) and all were retrieved (n = 0 not retrieved). Reports were assessed for eligibility (n = 125), with 110 full-text reports excluded, resulting in 15 studies included in the final review. Abbreviations: PRISMA, Preferred Reporting Items for Systematic Reviews and Meta-Analyses.

The database search identified 1,126 records. After removal of 245 duplicates, 881 unique records were screened by title and abstract. Of these, 756 were excluded as irrelevant or non-mRNA-based; 125 full texts were assessed; and 15 studies met inclusion criteria and were included in the qualitative synthesis (Figure 2).

### 3.4 Composition of included studies (n = 15)

- Seven preclinical studies (LNP optimisation, organ-selective delivery, saRNA/circRNA formats).
- Four translational studies (oncology and autoimmunity applications).
- Three early-phase clinical trials (therapeutic safety/dosing).
- One systematic review/meta-analysis providing mechanistic/regulatory context.

### 3.5 Data extraction and synthesis

Data were extracted into a structured matrix capturing study design, nanoparticle composition, RNA format, indication, outcomes, and translational status. Given rapid-review constraints, extraction was conducted by a single reviewer with an independent cross-check against indexed abstracts. Findings were synthesised narratively by theme (platform design, oncology, autoimmunity, genetic diseases, and translational barriers). Meta-analysis was not undertaken due to heterogeneity in designs and endpoints.

### 3.6 Limitations

This review was not registered in PROSPERO due to its cross-indication scope, but reporting was aligned with PRISMA-P principles for transparency. Single-reviewer screening and extraction may increase selection bias. Search documentation and structured extraction were used to support reproducibility.

## 4 Main findings/Thematic overview

### 4.1 Platforms and mechanistic enablers

LNPs dominate clinical translation, and the composition of ionisable lipids, helper lipids, and cholesterol analogues influences colloidal stability, apparent pKa, tissue tropism, and endosomal escape (Pastre et al., 2025; Paroor et al., 2025; Wu et al., 2024). In most settings, unmodified LNPs exhibit predominant hepatic accumulation; extrahepatic delivery is being pursued using organ-selective formulations and targeting strategies designed to alter protein corona composition, cellular uptake pathways, and biodistribution (Asthana et al., 2025; Alshehry et al., 2025; Han et al., 2026). These approaches should be described with mechanistic precision: passive uptake driven by biodistribution and biomolecular corona effects differs from receptor-mediated targeting achieved through ligand decoration and cell-specific binding.

Beyond LNPs, polymeric and hybrid nanoparticles (including lipopolyplexes) and exosome-inspired systems have been explored to improve targeting and to modulate immunostimulatory properties; however, endosomal escape remains a principal efficiency bottleneck, with low cytosolic release prompting lipid-phase engineering, fusogenic additives, and pH-responsive designs (Younis et al., 2025; Yang et al., 2025; Prazeres et al., 2025; Zwolsman et al., 2025). RNA formats also shape dose and durability: saRNA can reduce dose while sustaining expression, whereas circRNA may provide enhanced nuclease resistance and prolonged translation (Shahsavandi et al., 2024; Ren et al., 2025; Li et al., 2025; Mrksich et al., 2024; Ge et al., 2025). Materials innovation increasingly emphasises biodegradability; ester- or disulphide-containing ionisable lipids may accelerate clearance and reduce tissue retention while maintaining activity (Simonsen and Larsson, 2025; Lee et al., 2025; Alasmari et al., 2025). Thermostability and deployability remain central: lyophilised or solid-state formulations aim to reduce cold-chain dependence and improve access, although comparative stability data across platforms remain limited in the included evidence base (Gomi et al., 2023).

### 4.2 Oncology: immunostimulatory mRNA nanomedicines

Personalised neoantigen mRNA vaccination represents the most clinically mature therapeutic application. In KEYNOTE-942 (phase 2b), mRNA-4157/V940 plus pembrolizumab improved recurrence-free and distant metastasis-free survival compared with pembrolizumab alone (approximately 49% reduction in recurrence or death), supporting phase-3 programmes (Weber et al., 2024). In parallel, tumour-directed delivery strategies seek to enhance intratumoural mRNA expression and immune activation, including constructs encoding cytokines (e.g., IL-12, GM-CSF) and dendritic-cell–targeted formulations to improve antigen presentation and CD8^+^ T-cell expansion; most supporting evidence remains preclinical or early clinical (Floudas et al., 2025). Key barriers include efficient extrahepatic and tumour delivery, stromal penetration, and durability of response. Formulation strategies must balance potency with innate immune sensing to avoid excessive inflammation that may reduce efficacy or tolerability (Alasmari et al., 2025).

### 4.3 Autoimmunity: antigen-specific tolerance with mRNA nanoparticles

Tolerogenic mRNA delivery aims to induce antigen-specific immune regulation without global immunosuppression. Krienke et al. showed that nucleoside-modified mRNA-LNPs encoding autoantigens induced antigen-specific tolerance and ameliorated disease in experimental autoimmune encephalomyelitis (EAE) without systemic immunosuppression (Krienke et al., 2021). Related work supports engineering of spleen-targeted or tolerogenic LNPs, including formulations enriched with immunomodulatory lipids, to bias immune processing towards regulatory phenotypes (Gomi et al., 2023; Krienke et al., 2021). Design variables that influence tolerogenic outcomes include lipid pKa, nucleoside chemistry, and splenic tropism, which together determine innate activation and downstream immune programming. Translational priorities include clarifying durability under repeat dosing and defining clinically relevant endpoints for human autoimmune disease trials (Younis et al., 2025).

### 4.4 Genetic diseases: transient protein replacement and genome editing applications

Therapeutic mRNA can support transient protein replacement, with delivery route and tissue access determining feasibility. In cystic fibrosis, inhaled CFTR mRNA (ARCT-032) was reported as safe and well tolerated in phase 1 and has progressed to phase 2; early efficacy signals were described as mixed and dose-dependent, underscoring the need for adequately powered and sustained studies (Geller et al., 2024). For metabolic or hepatic indications, preclinical biodegradable LNPs have enabled *in vivo* expression of missing enzymes, indicating potential as an adjunct or alternative to enzyme replacement approaches (Richard et al., 2024). In cardiovascular or ischaemia models, circRNA-LNPs have been reported to prolong VEGF expression and support angiogenesis in preclinical settings, illustrating the potential of alternative RNA modalities where prolonged translation is desirable (Chai et al., 2025; Sayed et al., 2025).

Table 1 summarizes the evidence maturity (2020–2025) for nanoparticle-mediated therapeutic mRNA (non-vaccine) applications, highlighting where clinical translation is most advanced and where progress remains predominantly preclinical.

**TABLE 1:** Evidence profile of nanoparticle-mediated mRNA therapeutics beyond vaccines (2020–2025): domains, maturity, and anchor references.

Domain (therapeutic focus)	What the evidence mostly contains	Maturity (2020–2025)	Anchor examples
Oncology (immunostimulatory mRNA-NPs)	Personalised neoantigen vaccines; tumour immune reprogramming; delivery/tropism optimisation	Highest clinical maturity (phase 2b signal; phase-3 programmes initiated)	KEYNOTE-942 neoantigen mRNA + pembrolizumab (Weber et al., 2024); delivery for precision tumour therapy (Fei et al., 2024); antigen-based IO synthesis (Floudas et al., 2025)
Autoimmunity (tolerogenic mRNA-NPs)	Antigen-specific tolerance using nucleoside-modified mRNA-LNPs; splenic/immune targeting strategies	Strong preclinical, limited clinical	Non-inflammatory/tolerogenic mRNA-LNP in EAE (Krienke et al., 2021); tolerogenic LNP self-antigen delivery (Gomi et al., 2023)
Genetic/protein replacement (incl. lung)	Protein expression restoration (e.g., CFTR); biodistribution and route effects	Early clinical (signals emerging) plus enabling preclinical	Inhaled CFTR mRNA (phase 1 safety/tolerability; phase 2 ongoing) (Geller et al., 2024); PK/BD by lipid type and route (Ren et al., 2025)
Platform/mechanistic enablers	Organ-selective delivery, lipid SAR, endosomal escape mechanisms, formulation stability considerations	Rapidly expanding; key bottlenecks persist	Organ-selective LNP delivery *in vivo* (Zhang et al., 2024); endosomal escape bottleneck (Chatterjee et al., 2024); extrahepatic delivery challenges/opportunities (Simonsen, 2024); lipid pKa perspective (Simonsen and Larsson, 2025)

Abbreviations: BD, biodistribution; EAE, experimental autoimmune encephalomyelitis; IO, immuno-oncology; LNP, lipid nanoparticle; mRNA-NP, nanoparticle-mediated mRNA.

This table summarises the studies included in the qualitative synthesis, categorised by study type (preclinical, mechanistic, review, perspective, clinical), therapeutic indication, nanoparticle platform or formulation variable, administration route (where reported), primary biological or clinical endpoints, and specific translational contribution.The dataset spans oncology (including phase 2b clinical validation in melanoma), autoimmunity (antigen-specific tolerance in EAE models), genetic/protein replacement (early clinical inhaled CFTR mRNA), and platform-enabling mechanistic and structure–activity relationship (SAR) studies. Collectively, the studies define key translational determinants, including organ-selective targeting, route-dependent pharmacokinetics/biodistribution (PK/BD), ionisable lipid chemistry (tail length, unsaturation, apparent pKa), potency-immunogenicity balance, and endosomal escape efficiency (Table 2).

**TABLE 2:** Included evidence set (n = 15): study type, platform, route, primary readout, and relevance to therapeutic translation.

Ref.	Study type	Indication/Use-case	Platform/Variable	Route (as reported)	Primary readout(s)	Why it matters for this review
Fei et al. (2024)	Preclinical	Precision tumour therapy (lung metastasis model)	Tissue/cell-targeted LNP strategy	*In vivo* (reported)	Tumour/therapeutic response	Demonstrates extrahepatic/tumour-oriented delivery design
Simonsen (2024)	Review	Extrahepatic delivery	LNP strategies and constraints	—	—	Frames liver-tropism constraints and solution space
Zhang et al. (2024)	Preclinical	Organ-selective NA delivery	Organ-selective LNPs	*In vivo*	Organ-level delivery	Supports organ-selective targeting claims
Chatterjee et al. (2024)	Mechanistic perspective	Endosomal escape	LNP-mediated escape bottleneck	—	—	Establishes escape as a central efficiency limiter
Wang et al. (2025a)	Review	Endosomal escape control	Lipid-raft/phase concepts	—	—	Summarises formulation–escape design levers
Ren et al. (2025)	Preclinical PK/BD	Route dependence	Ionisable lipid type vs. PK/BD	IV, SC	PK/BD	Supports route selection and lipid chemistry effects
Li et al. (2025)	Preclinical	Efficacy vs. immunogenicity	mRNA vs. saRNA in LNPs	Intravitreal	Expression and immunogenicity	Demonstrates modality trade-offs (potency vs. innate sensing)
Mrksich et al. (2024)	Preclinical	Formulation SAR	Ionisable lipid tail length	*In vivo* (reported)	Delivery efficiency	Identifies SAR lever for potency/robustness
Ge et al. (2025)	Preclinical	Formulation SAR	Tail unsaturation in ionisable lipids	*In vivo* (reported)	Delivery and immunogenicity	Links lipid chemistry to potency–immunogenicity coupling
Simonsen and Larsson (2025)	Perspective	Formulation principle	Apparent pKa of ionisable lipids	—	—	Connects pKa to efficacy/escape
Lee et al. (2025)	Review/SAR	Design rules	Ionisable lipid SAR (siRNA/mRNA)	—	—	Extends transferable design principles
Gomi et al. (2023)	Preclinical	Autoimmunity (EAE)	Tolerogenic LNP self-antigen mRNA	*In vivo* (reported)	Disease scores/tolerance	Demonstrates antigen-specific tolerance
Krienke et al. (2021)	Preclinical (landmark)	Autoimmunity (EAE)	Non-inflammatory nucleoside-modified mRNA-LNP	*In vivo*	Tolerance	Proof-of-concept for tolerogenic mRNA therapeutics
Weber et al. (2024)	Clinical (phase 2b)	Melanoma (adjuvant)	Individualised neoantigen LNP-mRNA + pembrolizumab	Clinical	RFS/DMFS	Highest-level therapeutic clinical signal in this review
Geela et al. (2024)	Early clinical (phase 1 report)	Cystic fibrosis	Inhaled CFTR mRNA (ARCT-032)	Inhaled	Safety/tolerability	Anchors feasibility of lung delivery

Abbreviations: DMFS, distant metastasis-free survival; IV, intravenous; PK/BD, pharmacokinetics/biodistribution; RFS, recurrence-free survival; SAR, structure–activity relationship; SC, subcutaneous.

Table 3 provides a concise clinical translation snapshot of the therapeutic mRNA-nanoparticle programs highlighted in this review, emphasizing route feasibility, endpoint alignment, and near-term translational implications.

**TABLE 3:** Clinical translation snapshot (therapeutic mRNA-NP programmes highlighted in this review).

Program (ref.)	Indication	Platform/Cargo	Route	Phase (as cited)	Trial ID	Status	Key endpoint(s)	Key takeaway for clinicians/Researchers
mRNA-4157/V940 + pembrolizumab (Weber et al., 2024)	Resected high-risk melanoma (adjuvant)	Individualised neoantigen LNP-mRNA + anti-PD-1	Systemic	Phase 2b	NCT03897881	Active, not recruiting	RFS, DMFS	Clinically meaningful ↓ recurrence risk; supports phase-3 advancement
ARCT-032 (LUNAR®-CFTR mRNA) (Geller et al., 2024)	Cystic fibrosis	Nebulised LNP-mRNA (CFTR)	Inhaled	Phase 1 (reported) → Phase 2 (noted)	NCT06747858	Recruiting	Safety/tolerability (early); lung function/biomarkers (as available)	Feasibility of repeat-dose, non-invasive airway delivery; efficacy still consolidating
VX-522 (Vertex/Moderna)	Cystic fibrosis (non-modulator eligible genotypes)	Inhaled mRNA-LNP (CFTR)	Inhaled	Phase 1/2	NCT05668741	Recruiting	Safety/tolerability; exploratory efficacy	Confirms field move toward inhaled LNP-mRNA CFTR replacement; watch for dose-limiting tolerability signals
mRNA-3927	Propionic acidemia	Systemic LNP-mRNA (enzyme replacement)	IV	Phase 1/2	NCT04159103	Recruiting	Safety; PK/PD biomarkers; metabolic decompensation (as applicable)	“Intracellular protein replacement” via LNP-mRNA is clinically testable; endpoints rely heavily on PD/biomarkers early
mRNA-3705	Isolated methylmalonic acidemia (MUT deficiency)	Systemic LNP-mRNA (enzyme replacement)	IV	Phase 1/2	NCT04899310	Active, not recruiting	Safety; plasma MMA reduction (PD/efficacy)	Shows how rare-disease mRNA-LNP trials anchor on biomarker efficacy + tolerability over repeated IV dosing
ARCT-810 (LUNAR®-OTC)	Ornithine transcarbamylase deficiency	Systemic LNP-mRNA (OTC)	IV	Phase 2a	NCT06488313	Recruiting	Safety/PD; ammonia control (as applicable)	Tests whether repeated LNP-mRNA dosing can achieve clinically meaningful metabolic control in urea-cycle disorders
mRNA-2752 (triplet cytokine/ligand) ± durvalumab	Advanced solid tumours/lymphoma (IT)	LNP-mRNA encoding OX40L/IL-23/IL-36γ	Intratumoral	Phase 1	NCT03739931	Completed (study completion listed)	Safety; immune PD markers; response (exploratory)	Illustrates IT mRNA-LNP as an “in situ immune-engineering” approach; translational readouts hinge on tumour immune modulation
BNT111 (FixVac) ± cemiplimab	Advanced melanoma (PD-1/PD-L1 refractory/relapsed)	RNA-lipoplex cancer immunotherapy	Systemic	Phase 2	NCT04526899	Completed	ORR/PFS (trial-specific)	Provides comparator “non-LNP RNA nanoparticle” oncology experience; useful for contextualising delivery platform vs. clinical activity

Trial status reflects the most recent listing on ClinicalTrials.gov at time of manuscript revision (last update postings include 3 Dec 2025 for NCT03897881; 9 Feb 2026 for NCT05668741; 22 Jan 2026 for NCT04159103; 20 Jan 2026 for NCT04899310).

### 4.5 Challenges and Open Questions

Therapeutic mRNA delivery beyond vaccinology is constrained by a recurring set of translational barriers that are incompletely resolved in current evidence. Extrahepatic targeting remains difficult because biodistribution is dominated by liver uptake for many conventional LNP designs (Simonsen, 2024; Zhang et al., 2024). The relative contribution of passive biodistribution, biomolecular corona formation, and ligand-driven receptor targeting should be specified when interpreting ‘targeting’ claims to avoid conflating mechanisms.

Endosomal escape is widely recognised as a limiting step for functional delivery, motivating lipid design strategies that modify membrane interactions and phase behaviour (Chatterjee et al., 2024), (Wang X. et al., 2025), (Simonsen and Larsson, 2025). For chronic indications requiring repeat dosing, innate immune sensing and anti-carrier immune responses may reduce durability and tolerability; nucleoside modification and immunologically ‘silent’ designs are relevant levers, but comparative human data remain sparse (Krienke et al., 2021), (Gomi et al., 2023), (Ge et al., 2025). PEG-associated hypersensitivity has been reported in LNP contexts and supports interest in PEG-sparing alternatives; however, prevalence and risk factors should be described cautiously and anchored to cited evidence.

Manufacturing and stability remain decisive for clinical translation and equitable access. Thermostable and lyophilised presentations may reduce cold-chain dependence, but formulation-dependent changes in particle size distribution, encapsulation efficiency, potency, and impurity profiles require standardised reporting and quality-by-design control (Mrksich et al., 2024), (Gomi et al., 2023). Regulatory pathways must also align with therapeutic endpoints, long-term monitoring, and repeat-dose considerations, particularly for genetic disease applications and any programmes involving genome editing.

Table 4 consolidates the shared translational bottlenecks that repeatedly constrain therapeutic mRNA nanoparticle development beyond vaccines, and links each barrier to actionable engineering solution classes and a minimum reporting set needed for reproducibility and interpretability.

**TABLE 4:** Cross-cutting translational barriers and engineering solutions in nanoparticle-mediated mRNA therapy: what to report.

Barrier (cross-cutting)	Why it limits translation	Practical solution classes (examples)	Minimum reporting for Q1 rigour	Key supporting refs
Extrahepatic targeting	Default liver tropism limits disease breadth	Organ-selective LNPs; composition/charge tuning; route optimisation	Quantitative tissue BD and target-cell identification	Fei et al. (2024), Simonsen (2024), Zhang et al. (2024)
Endosomal escape	Major efficiency bottleneck for cytosolic delivery	Ionisable lipid tuning; membrane/phase engineering; raft-focused strategies	Escape assay linked to functional protein output	Chatterjee et al. (2024), Wang et al. (2025a), Simonsen and Larsson (2025)
Repeat-dose immunogenicity	Chronic indications require re-dosing; innate sensing may erode efficacy	Nucleoside modification; immunologically ‘silent’ designs; lipid SAR optimisation	Cytokines, complement activation, anti-component antibodies; durability	Ge et al. (2025), Gomi et al. (2023), Krienke et al. (2021)
Potency–immunogenicity trade-off	Higher expression may coincide with stronger innate activation	Format selection (mRNA vs. saRNA); lipid tail/SAR tuning	Head-to-head potency and immune markers under matched dosing	Li et al. (2025), Mrksich et al. (2024), Ge et al. (2025)
Route dependence	Route alters PK/BD and immune exposure	IV vs. SC vs. local vs. inhaled route selection driven by target organ	PK/BD by route and exposure–response relationship	Ren et al. (2025), Geller et al. (2024)
Design rationalisation (pKa/SAR)	Empirical design limits reproducibility and scale-up	Apparent pKa targeting; SAR frameworks	Full formulation disclosure; pKa/ionisation rationale; CQAs	[27], [28]

Abbreviations: BD, biodistribution; CQA, critical quality attribute; PK, pharmacokinetics; SAR, structure–activity relationship.

### 4.6 Solid lipid nanoparticles (SLN) for mRNA delivery: opportunities beyond immunization, and persistent hurdles

Solid lipid nanoparticles (SLNs) are colloidal carriers built from physiologically tolerated solid lipids (often triglycerides/waxes) stabilized by surfactants, offering high physical stability and established manufacturing routes (e.g., high-pressure homogenization) that are familiar to pharmaceutical development (Sayed et al., 2025; Müller et al., 2000; Mehnert and Mäder, 2001; Schwarz et al., 1994). Their appeal for therapeutic mRNA extends from (i) a solid lipid matrix that can protect labile cargo during handling/storage and (ii) the prospect of PEGylation/surface engineering to tune circulation and tissue exposure. However, translating SLNs from small-molecule encapsulation to large, polyanionic mRNA places stringent constraints on loading, structural stability, and endosomal escape issues that differ materially from conventional ionizable LNPs used in vaccines (Sayed et al., 2025; Müller et al., 2000; Mehnert and Mäder, 2001; Schwarz et al., 1994).

#### 4.6.1 Recent advances that may enable therapeutic use beyond immunization

Ionizable-lipid incorporated SLNs (iSLNs/PEG-iSLNs). A key step-change has been the move from “neutral” SLNs toward SLNs doped with ionizable/cationic lipids to improve electrostatic complexation, cellular uptake, and critically endosomal escape, while maintaining the formulation’s solid-lipid stability features (Müller et al., 2000; Mehnert and Mäder, 2001; Schwarz et al., 1994). Recent work describing PEGylated, ionizable-lipid–incorporated SLNs for mRNA and pDNA delivery supports the concept that SLN-like matrices can be engineered into functional gene delivery systems rather than passive depots. “Second-generation” solid lipid systems: nanostructured lipid carriers (NLCs) and defect-engineered matrices (Gómez-Aguado et al., 2020). A recurring limitation of classical SLNs is the tendency of highly crystalline matrices to exclude (“squeeze out”) payload on storage (Joshi et al., 2025). NLCs (solid + liquid lipid blends) intentionally introduce lattice imperfections to increase loading capacity and reduce expulsion risk, and are increasingly discussed as a pragmatic evolution path for nucleic acid cargo where the “solid core” concept is retained but crystallinity is moderated (Schwarz et al., 1994). Process innovation for tighter control (microfluidics, continuous processing) (Gómez-Aguado et al., 2020). Advanced preparation approaches (including microfluidic strategies) are being explored to reduce batch-to-batch variability, control size/PDI, and potentially enable continuous manufacture important if SLN-mRNA is to compete with LNPs on reproducibility (Joshi et al., 2025). Collectively, these advances suggest a plausible route for SLN-type carriers into repeat-dose therapeutic domains (protein replacement, local delivery, chronic indications) provided that loading/release and tolerability barriers can be solved at clinically relevant dose intensities.

#### 4.6.2 Critical appraisal: challenges and open questions (aligned to “challenges and open questions”)

Encapsulation constraints for large RNAs (loading vs. functionality trade-off): Unlike hydrophobic small molecules that partition readily into solid lipid matrices, mRNA’s size and polyanionic character complicate true “encapsulation” in a crystalline core. Practically, many SLN formulations rely on (a) surface adsorption/complexation using cationic components or (b) incorporation of ionizable lipids to form electrostatic complexes (Sayed et al., 2025; Müller et al., 2000). This can constrain achievable mRNA payload, increase sensitivity to ionic strength/serum proteins, and may raise cationic-lipid linked tolerability concerns at therapeutic dosing. The field still lacks consensus on what constitutes robust, scalable, high-EE mRNA loading in an SLN architecture without reverting to conventional LNP designs (Mehnert and Mäder, 2001).

Polymorphic transitions and their impact on release and stability: A central SLN-specific risk is lipid polymorphism: many triglycerides transition from metastable α/β′ forms to the more stable β form during storage, which can alter lattice packing, promote payload expulsion, and change release kinetics (often unpredictably) (Schwarz et al., 1994). For mRNA (where both integrity and timing of cytosolic availability matter), such transitions could shift performance between batches or over shelf-life unless tightly controlled (lipid selection, stabilizers, NLC-type matrices, and robust QC for polymorphic state) (Gómez-Aguado et al., 2020).

Scalability, sterilization, and “GMP reality: While high-pressure homogenization is attractive for scale-up, mRNA adds sensitivity to heat/shear, and SLN size distributions can drift with process parameters raising comparability risks across development stages (Müller et al., 2000). Sterility is also non-trivial: terminal sterilization (heat, irradiation) may perturb lipid matrices, while sterile filtration can be challenged by nanoparticle size, filter fouling, and adsorption losses (Mehnert and Mäder, 2001). These issues are increasingly recognized across lipid-based nanomedicines and should be explicitly considered early for SLN-mRNA CMC plans (aseptic processing strategy, validated hold times, container/closure interactions) (Schwarz et al., 1994).

Repeat-dose tolerability and immunological liabilities (especially surface chemistries): Chronic or repeat-dose therapeutic use places higher emphasis on innate immune activation, complement activation, and accelerated blood clearance phenomena (Mehnert and Mäder, 2001). PEGylation can stabilize dispersions and prolong circulation, but PEG is also linked to anti-PEG antibodies, complement activation, and hypersensitivity signals in lipid nanoparticle settingsri sks that may be amplified under repeated exposure (Schwarz et al., 1994). For SLN-mRNA (which is often positioned for chronic indications), the immunology of both the surface polymer and any cationic/ionizable lipids used to bind mRNA becomes a primary “open question,” and may motivate alternative stealth coatings (or dosing regimens) in later-stage development (Gómez-Aguado et al., 2020).

Bottom line for the “Challenges and Open Questions” section. SLNs/NLCs represent a credible “adjacent” platform to LNPs with potential advantages in physical stability and manufacturing familiarity, and recent ionizable-lipid-enabled SLN designs strengthen the case for therapeutic (non-vaccine) use. Yet, the platform remains gated by (1) true high-capacity mRNA loading, (2) control of polymorphism-driven instability and release variability, (3) sterile GMP manufacture without damaging the lipid matrix or RNA, and (4) repeat-dose tolerability under clinically relevant dosing schedules (Schwarz et al., 1994; Gómez-Aguado et al., 2020; Joshi et al., 2025; Tricco et al., 2018).

## 5 Discussion

Programmable mRNA cargoes combined with nanoparticle delivery have demonstrated scalability and acceptable safety in population-scale vaccination, and therapeutic development is extending to individualised cancer vaccination, antigen-specific immune tolerance, and transient protein replacement without genomic integration (Fei et al., 2024; Wang X. et al., 2025; Wang Y. et al., 2025; Yu et al., 2025). Across the included evidence, preclinical support is substantial, early clinical evidence is emerging most clearly in oncology and cystic fibrosis while late-stage therapeutic trials remain limited. Statements regarding overall evidence levels should be interpreted in the context of the small number of included studies and the heterogeneity of endpoints.

Biodistribution remains constrained by hepatic sequestration for many LNP designs, despite progress in lipid chemistry, ligand decoration, route optimisation, and strategies intended to modulate corona formation and cellular uptake (Asthana et al., 2025). Endosomal escape remains a major barrier, and reported cytosolic release efficiencies are typically low, motivating rational lipid design and modulation of membrane phase behaviour (Prazeres et al., 2025). Safety and immunogenicity considerations are central for therapeutic, repeat-dose applications. Claims regarding post-vaccine myocarditis and PEG-related hypersensitivity should be supported by appropriate clinical safety literature; such evidence is not clearly represented within the current reference set and therefore requires careful citation alignment.

Access and stability remain practical constraints. Lyophilised or solid formulations and control of lipid impurities may improve long-term stability and reduce cold-chain dependence, but reproducible manufacturing depends on robust characterisation and reporting of critical quality attributes and performance metrics (Mrksich et al., 2024). The field is also adopting data-driven formulation and process optimisation (including microfluidic production and computational design), and near-term milestones include phase-3 neoantigen programmes and broader exploration of organ-selective delivery for lung and liver disease (Simonsen and Larsson, 2025).

For clinicians, key implications include awareness of emerging adjuvant melanoma mRNA programmes and the expansion of early trials in cystic fibrosis and metabolic disorders, coupled with careful monitoring for immune-mediated adverse events in settings where repeat dosing is required. For researchers and developers, priorities include organ-selective and biodegradable lipid systems, standardised assays for endosomal escape linked to functional expression, transparent PK/PD reporting, and stability-focused formulations compatible with low-resource settings under quality-by-design control (Gomi et al., 2023; Weber et al., 2024; Floudas et al., 2025; Krienke et al., 2021; Geller et al., 2024; Richard et al., 2024; Chai et al., 2025; Sayed et al., 2025).

## 6 Conclusion

Nanoparticle-mediated mRNA therapy is progressing beyond infectious-disease vaccines into oncology, autoimmunity, and genetic medicine. Platform maturation including biodegradable and organ-selective lipids, saRNA/circRNA modalities, and stability-oriented formulations addresses longstanding delivery and storage constraints. To support durable therapeutic impact, the field now requires disease-specific trials, standardised reporting of biodistribution and endosomal escape, and regulatory pathways aligned with chronic use and repeat dosing. Achieving these objectives would strengthen the evidence base for mRNA-nanoparticle therapeutics across precision immunotherapy and molecular medicine.

Table 5 collates representative, high-signal examples (2020–2025) showing how nanoparticle-enabled mRNA has moved beyond infectious-disease vaccines into (i) clinically anchored oncology, (ii) mechanistically compelling immune tolerance, and (iii) organ/CMC innovations that expand feasible targets, dosing strategies, and global deployability.

**TABLE 5:** Representative studies of nanoparticle-mediated mRNA delivery beyond vaccines (2020–2025).

Year	Indication	Platform/Cargo	Model/Phase	Key finding	Source
2023–2024	Melanoma (adjuvant)	LNP-mRNA individualised neoantigen vaccine (mRNA-4157/V940) + pembrolizumab	Phase 2b	∼49% lower risk of recurrence or death vs. pembrolizumab alone; improved DMFS	Pastre et al. (2025), Alshehry et al. (2025), Yang et al. (2025)
2021	Autoimmune neuroinflammation	Tolerogenic LNP-mRNA encoding myelin antigens	EAE (mouse)	Antigen-specific tolerance without systemic immunosuppression	Yang et al. (2025), Zwolsman et al. (2025), Li et al. (2025)
2024–2025	Cystic fibrosis	Inhaled LNP-CFTR mRNA (ARCT-032)	Phase 1 → Phase 2	Acceptable safety/tolerability; phase-2 evaluation ongoing	Mrksich et al. (2024), Ge et al. (2025), Lee et al. (2025)
2023	Extrahepatic targeting (lung)	Charged-helper-lipid LNPs	Multi-cell lung models	Altered tropism and transcriptional response enabling lung delivery	Alasmari et al. (2025), Gomi et al. (2023), Weber et al. (2024)
2024–2025	Endosomal escape	LNP design/raft engineering	Reviews/mechanistic	Endosomal escape remains limiting; strategies aim to improve cytosolic release	Prazeres et al. (2025), Shahsavandi et al. (2024), Li et al. (2025)
2024–2025	Stability	Lyophilised/solid mRNA-LNPs; impurity-controlled lipids	Formulation studies	Extended stability towards room temperature	Gomi et al. (2023), Krienke et al. (2021), Geller et al. (2024)
2023–2025	Biodegradable lipids	Disulphide/ester-linked ionisable lipids	Libraries/preclinical	Faster clearance with maintained activity	Weber et al. (2024), Geller et al. (2024), Richard et al. (2024)

## Data Availability

No new primary datasets were generated in this study. This rapid review was based on publicly available peer-reviewed articles and registered clinical trials. All studies and trial records included in the review are cited in the manuscript.
